# Myeloid Infection Links Epithelial and B Cell Tropisms of Murid Herpesvirus-4

**DOI:** 10.1371/journal.ppat.1002935

**Published:** 2012-09-20

**Authors:** Bruno Frederico, Ricardo Milho, Janet S. May, Laurent Gillet, Philip G. Stevenson

**Affiliations:** Division of Virology, Department of Pathology, University of Cambridge, Addenbrookes Hospital, Cambridge, United Kingdom; University of California-Berkeley, United States of America

## Abstract

Gamma-herpesviruses persist in lymphocytes and cause disease by driving their proliferation. Lymphocyte infection is therefore a key pathogenetic event. Murid Herpesvirus-4 (MuHV-4) is a rhadinovirus that like the related Kaposi's Sarcoma-associated Herpesvirus persists in B cells *in vivo* yet infects them poorly *in vitro*. Here we used MuHV-4 to understand how virion tropism sets the path to lymphocyte colonization. Virions that were highly infectious *in vivo* showed a severe post-binding block to B cell infection. Host entry was accordingly an epithelial infection and B cell infection a secondary event. Macrophage infection by cell-free virions was also poor, but improved markedly when virion binding improved or when macrophages were co-cultured with infected fibroblasts. Under the same conditions B cell infection remained poor; it improved only when virions came from macrophages. This reflected better cell penetration and correlated with antigenic changes in the virion fusion complex. Macrophages were seen to contact acutely infected epithelial cells, and cre/lox-based virus tagging showed that almost all the virus recovered from lymphoid tissue had passed through lysM^+^ and CD11c^+^ myeloid cells. Thus MuHV-4 reached B cells in 3 distinct stages: incoming virions infected epithelial cells; infection then passed to myeloid cells; glycoprotein changes then allowed B cell infection. These data identify new complexity in rhadinovirus infection and potentially also new vulnerability to intervention.

## Author Summary

Rhadinoviruses cause lymphocytic cancers. Their infection of lymphocytes is therefore an important therapeutic target. How this occurs is unclear. One prevalent hypothesis has been that virions directly infect lymphocytes when they enter new hosts. Here we show that host entry by Murid Herpesvirus-4, a close relative of the Kaposi's Sarcoma-associated Herpesvirus, is an epithelial rather than a lymphocyte infection: the mucosal lymphoid colonization typical of acute infectious mononucleosis only occurred later. Macrophages were closely associated with the acutely infected epithelium, and most if not all of the virus reaching B cells showed evidence of previous myeloid cell infection. Macrophage-derived virions showed a greatly enhanced capacity for lymphocyte infection that was associated with antigenic changes in the viral fusion proteins. Thus host colonization required epithelial and myeloid infections before there was lymphocyte infection. The implication is that each of these infection events could be independently targeted to limit viral persistence.

## Introduction

Herpesviruses are among the most prevalent of all persistent pathogens. Thus even when disease is individually rare, the total burden in populations is large. The difficulty of eliminating latent viral genomes makes latency establishment an important target for infection control. Gamma-herpesviruses persist in lymphocytes. Epstein-Barr virus (EBV) infects B cells better than epithelial cells *in vitro*, and prominently colonizes tonsillar B cells during acute infectious mononucleosis [1]. Thus EBV [2] and the Kaposi's Sarcoma-associated Herpesvirus (KSHV) [3] have been proposed to infect tonsillar B cells directly after oral host entry. However infectious mononucleosis post-dates EBV host entry by at least a month [4]. Therefore the infection seen at that time may correspond to host exit rather than entry, and vaccination to prevent B cell infection failed to reduce EBV seroconversion rates [5].

One barrier to understanding gamma-herpesviruses solely through EBV and KSHV is that their narrow species tropisms limit *in vivo* analysis. Experimentally accessible gamma-herpesviruses such as Murid Herpesvirus-4 (MuHV-4) [6]–[9] consequently provide an important source of information. MuHV-4 is closely related to KSHV [10], [11]. Like EBV and KSHV it persists in B cells [12]. It also infects myeloid cells [13]. Most experimental infections have delivered MuHV-4 intranasally to mice under general anesthesia; aspirated virions then infect lung epithelial cells [14]. The detection by PCR of replication-deficient viral DNA from flow cytometrically sorted lung B cells in this setting led to the idea that B cells are a direct infection target [15], [16]. However viral DNA^+^ B cells were not detected in lymphoid organs, and adsorbed inoculum debris was not excluded as the source of viral DNA. A further caveat to drawing general conclusions from lung infection is that MuHV-4 inhaled without anesthesia does not reach this site: it replicates just in the nose [17] before following a CD11c-dependent route to lymphoid tissue [18].

Our understanding of human herpesvirus infections is based largely on analysis *in vitro*. A key task with MuHV-4 is therefore to relate *in vitro* tropisms to host colonization. Fibroblast-propagated MuHV-4 efficiently infects mice [17], [19] yet like KSHV seems to infect B cells poorly: despite reports of MuHV-4 infected B cell lines [20], [21] and phenotypic changes in virus-exposed B cells [22], [23], efficient B cell infection has not been demonstrated. MuHV-4 depends on heparan sulfate (HS) to infect adherent cells [24], and poor B cell infection by MuHV-4 and KSHV has been attributed to B cells lacking HS [25]. However infection was not convincingly demonstrated even when B cell HS expression increased. Therefore the barriers to B cell infection remain ill-defined.

Here we found no evidence for direct mucosal B cell infection by MuHV-4 entering the upper respiratory tract. Host entry was instead an epithelial infection. This corresponded to *in vitro* B cell infection showing binding and post-binding blocks. Unlike B cell infection, myeloid infection was limited only by binding and worked well by co-culture with infected fibroblasts. B cell infection improved only when virions came from myeloid cells. These virions showed a constitutive triggering of entry-associated changes in gB and gH. Myeloid cells were closely associated with the acutely infected epithelium, and cre/lox virus marking showed that most of the virus reaching lymphoid tissue had passed through cells expressing CD11c and myeloid-specific lysozyme (lysM). Thus we propose that rhadinoviruses entering new hosts infect epithelial cells, then myeloid cells, and only then B cells.

## Results

### MuHV-4 infects the nasal-associated lymphoid tissue only as a secondary event in host colonization

**Figure 1:**
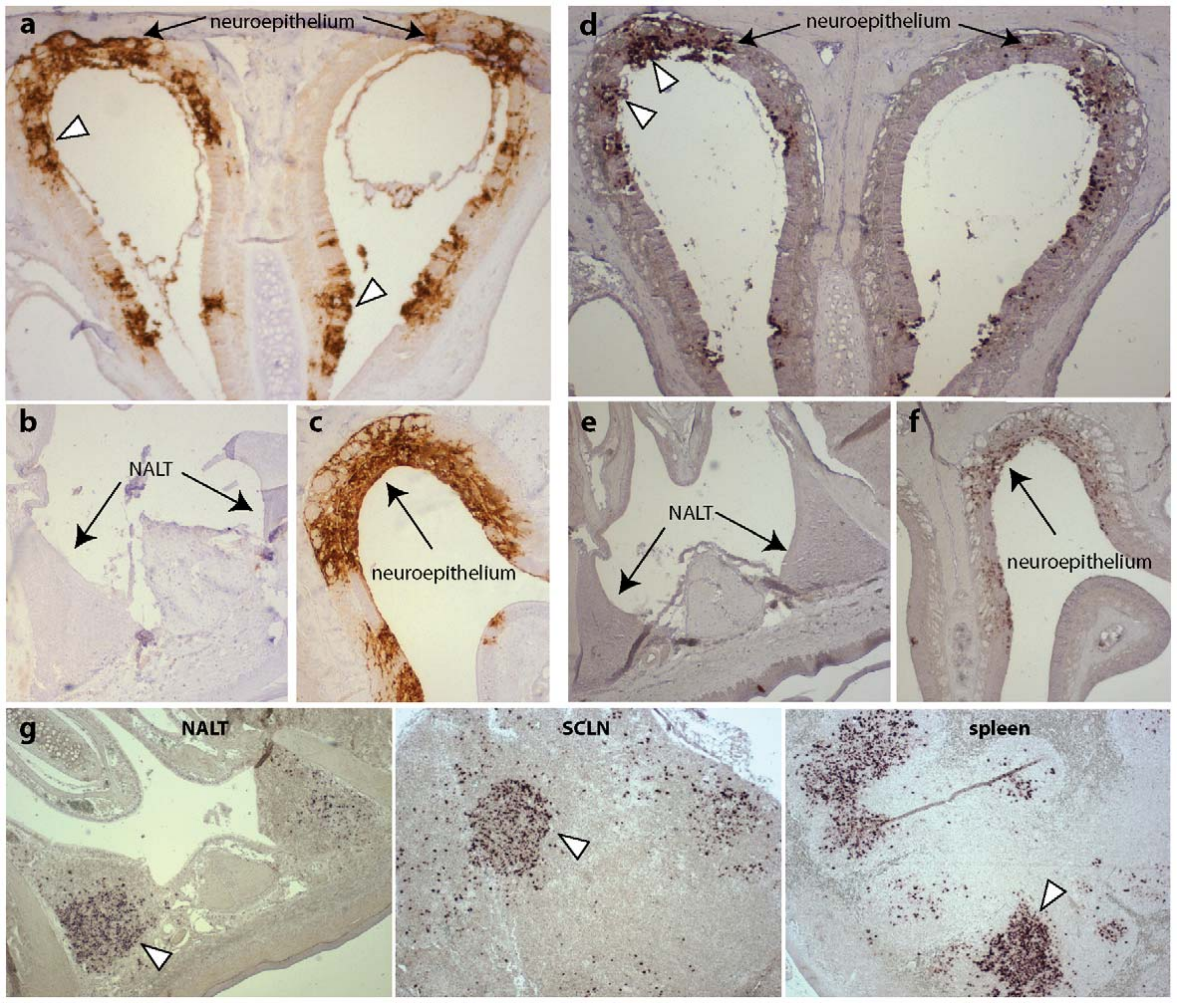
Detection of MuHV-4 infection in the upper respiratory tract. **a.** C57BL/6 mice were infected with MuHV-4 (3×10^4^ p.f.u. in 5 µl) and 6 days later examined by immunostaining nasal sections with a MuHV-4-specific polyclonal rabbit serum (brown). Arrowheads show examples of infected olfactory neuroepithelium. This and subsequent images are each representative of at least 3 mice examined. **b.** In contrast to the neuroepithelium, the nasal-associated lymphoid tissue (NALT) did not stain for viral antigens. **c.** Neuroepithelium on the same slide as the NALT in **b** stained strongly. **d.** Adjacent sections were assayed for viral miRNA/tRNA expression by *in situ* hybridization. Arrowheads show examples of infected olfactory neuroepithelium. **e.** No hybridization signal was observed in the NALT. **f.** Neuroepithelium on the same slide as the NALT was miRNA/tRNA^+^. **g.** At 14 days after virus inoculation (3×10^4^ p.f.u. in 5 µl), NALT, superficial cervical lymph node (SCLN) and spleen sections were examined for viral miRNA/tRNA expression by *in situ* hybridization. All were positive. Arrowheads show examples of infected cells.

MuHV-4 is non-infectious orally, but readily infects via the upper respiratory tract [17]. The murine nasal-associated lymphoid tissue (NALT) is analogous to human tonsils [26] and provides a potential target for lymphotropic viruses entering the nasopharynx. To determine whether MuHV-4 targets the NALT, we allowed unanesthetized mice to inhale virus and visualized infection 6 days later by immunostaining with a polyclonal, MuHV-4-specific rabbit serum (Fig. 1a–c). Lytic antigens were abundant in the olfactory neuroepithelium (Fig. 1a) but absent from the NALT and its overlying epithelium (Fig. 1b). Most of the neuroepithelium is anterior to the NALT, and so potentially more accessible, but even when neuroepithelial infection was evident in the same histological section (Fig. 1c) the NALT lacked viral antigens.

Our immune serum predominantly recognizes MuHV-4 lytic antigens [27], so it remained possible that the NALT was latently infected. We tested this by *in situ* hybridization for the viral tRNA/miRNAs that are abundantly expressed in latently infected splenic B cells [28]. Again neuroepithelial infection was readily identifiable (Fig. 1d) but NALT infection was not (Fig. 1e), even when neuroepithelial cells in the same histological section were tRNA/miRNA^+^ (Fig. 1f). At 14 days post-infection viral tRNA/miRNA expression was abundant in the NALT (Fig. 1g). However this post-dates the virus spread and amplification associated with infectious mononucleosis [7], and infection was accordingly abundant also in lymph nodes and the spleen (Fig. 1g). Thus the primary i.n. infection was epithelial, and NALT infection did not occur until there was systemic virus spread.

### Detecting B cell infection by eGFP expression

To understand why the NALT was not acutely infected we tested virions for their capacity to infect B cells. A key point was to reliably identify B cell infection. Viral tRNA/miRNA detection is not easily combined with staining for cell type-specific markers, and while some infected B cells express ORF73 and M2 [29], little ORF73 seems to be made [30] and M2 expression is unlikely to be universal. We therefore used an intergenic EF1α promoter to express constitutively viral eGFP (Fig. S1). Mice infected with EF1α-eGFP^+^ MuHV-4 showed eGFP expression in 1.7% of CD19^+^ lymph node B cells, consistent with PCR-based estimates of wild-type virus loads [29], [31], [32]; this virus also labelled more convincingly than did HCMV IE1-eGFP^+^ MuHV-4 A20 B cells over-expressing the HS carrier syndecan-1. It therefore provided a good basis for detecting B cell infection.

### B cell infection remains poor regardless of virion HS dependence

**Figure 2:**
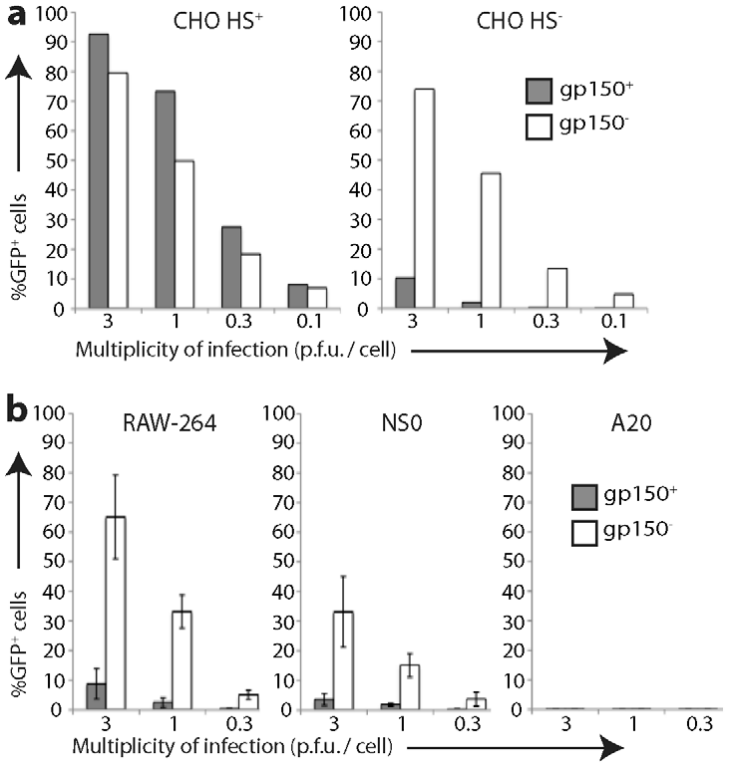
Infection of different cell types by gp150^+^ and gp150^−^ MuHV-4. **a.** HS^+^ and HS^−^ CHO-K1 cells were exposed to gp150^+^ and gp150**^−^** versions of EF1α-eGFP MuHV-4 for 18 h. 20,000 cells were then assayed for viral eGFP expression by flow cytometry. Equivalent protein content of the gp150^+^ and gp150^−^ virus stocks was confirmed by Coomassie staining (Fig. S2). **b.** RAW-264, NS0 and A20 cells were infected and analysed as in **a**. Bars show mean ± SD of 3 replicate experiments. Gp150^−^ virions infected RAW-264 and NS0 cells significantly better than did gp150^+^ virions (p<0.01 by Student's 2-tailed t test).

We next used EF1α-eGFP^+^ MuHV-4 to compare the infectibility of different cell types. For consistency with studies using other read-outs [20], [21], [24], [33] we tested CHO-K1 epithelial cells, RAW-264 myeloid cells, and NS0 and A20 B cells, exposing each to cell-free virions and assaying infection 18 h later by flow cytometry (Fig. 2). A particular question was how far cellular HS expression limits infection. MuHV-4 HS dependence is due in part to the inhibitory effect of its gp150, as gp150 null mutants are much less HS-dependent in binding and infection than the wild-type [24]. We therefore compared infection by gp150^+^EF1α-eGFP^+^ and gp150^−^EF1α-eGFP^+^ virions.

The gp150^−^ and gp150^+^ virions infected HS^+^ CHO epithelial cells similarly. The gp150^−^ virions infected HS^−^ CHO cells much better - approximately 30-fold fewer virions than wild-type gave an equivalent number of eGFP^+^ cells (Fig. 2a, 2b). They also infected RAW-264 monocytes better (Fig. 2c), arguing that poor HS expression limits myeloid infection. In contrast, both gp150^−^ and gp150^+^ virions infected A20 B cells poorly (<1% eGFP^+^ at 10 p.f.u./cell). The B cell lines WEHI-231 and BCL-1 also showed <1% eGFP expression after exposure (10 p.f.u./cell, 18 h) to gp150^+^EF1α-eGFP^+^ or gp150^−^EF1α-eGFP^+^ virions (data not shown). Therefore poor B cell infection could not be explained simply by a lack of HS. NS0 myeloma cells were infected better than A20, WEHI-231 or BCL-1, particularly by gp150^−^ virions, but remained less infectible than RAW-264 or HS^−^ CHO cells. Therefore all B cell-derived lines showed an infection block beyond poor HS expression.

### HS expression by different cell types

**Figure 3:**
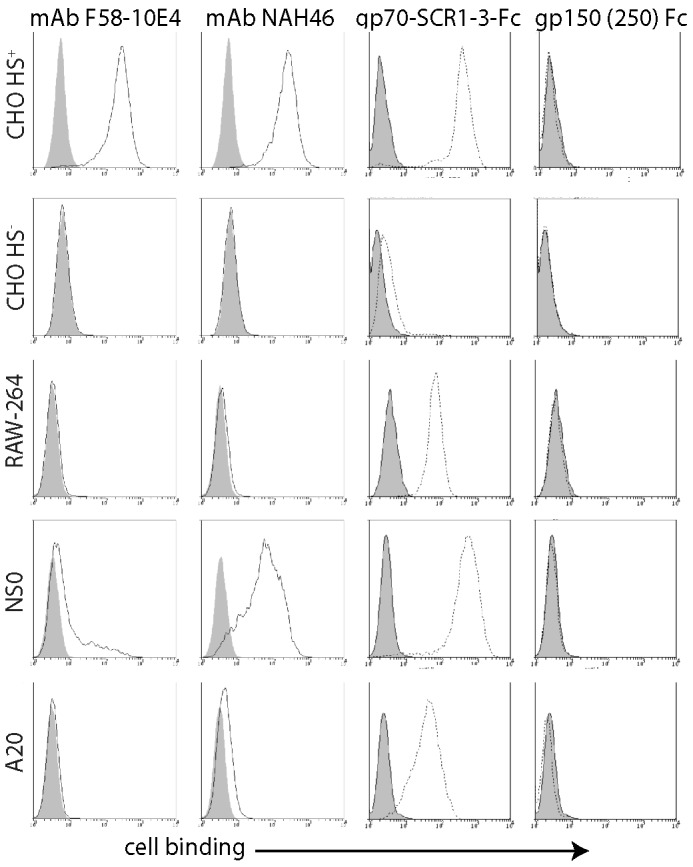
Flow cytometric assay of cell surface HS expression. Cells were plated overnight onto Petri dishes, then detached without trypsin and stained with HS-specific mAbs (F58-10E4, NAH46) or with the HS binding N-terminal 3 SCR domains of the virion gp70 linked to human IgG-Fc. The N-terminal 250 amino acids of gp150 linked to IgG-Fc provided a negative control. Open histograms show specific staining; filled histograms show staining by secondary antibody alone. Equivalent data were obtained in a repeat experiment.

We used flow cytometry to quantitate cellular HS display (Fig. 3). MAb F58-10E4 recognizes a sulfation-dependent epitope [34], while mAb NAH46 recognizes a sulfation-independent epitope [35]. Both recognize most forms of HS because it typically shows partial sulfation [36]. Neither mAb stained HS^−^ CHO cells, nor showed more than minimal staining of RAW-264 and A20 cells. NAH46 strongly stained HS^+^ CHO cells and NS0 cells, whereas F58-10E4 stained HS^+^ CHO cells strongly and NS0 cells only weakly. Therefore CHO cells expressed partially sulfated HS; NS0 cells expressed largely unsulfated heparan; and A20 and RAW-264 cells expressed little of either form.

What HS forms MuHV-4 binds to are unknown. We therefore measured functionally relevant HS display by staining cells with an Fc fusion of the viral gp70 HS binding domains (gp70-SCR1-3-Fc) [33] (Fig. 3). An Fc fusion of the MuHV-4 gp150 residues 1–250, which does not detectably bind to cells [37], provided a negative control. Gp70-SCR1-3-Fc binding was more sensitive than mAb binding, possibly because it binds to a wider range of HS modifications, but like mAb NAH46 it bound HS^+^ CHO and NS0 cells well, RAW-264 and A20 cells less well, and HS^−^ CHO cells hardly at all. Thus NS0 cells were infected poorly despite displaying HS for virus binding.

### HS interactions determine virion binding but not infection

**Figure 4:**
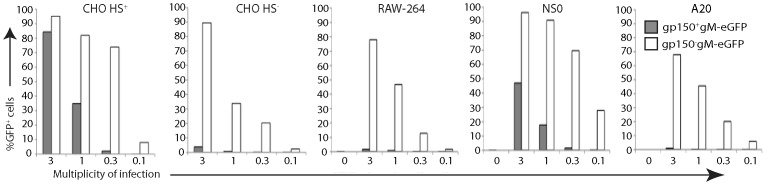
Binding of different cell types by gp150^+^ and gp150^−^ virions. Cells were exposed to gp150^+^ and gp150**^−^** versions of MuHV-4 with eGFP linked to the cytoplasmic tail of gM (gM-eGFP) (4 h, 37°C), then washed ×3 in PBS, and 20,000 cells assayed for virion binding/uptake by flow cytometry of eGFP fluorescence. Equivalent total protein content of the gp150^+^ and gp150^−^ virus stocks was confirmed by Coomassie staining, and equivalent eGFP content was confirmed by immunoblotting (Fig. S3). Gp150 disruption significantly increased virus binding to all cell types (p<10^−5^ by Chi-squared test, comparing the relative proportions of eGFP^+^ and eGFP^−^ cells over at least 3 virus dilutions). Similar data were obtained in a repeat experiment.

We next tested cell binding by virions, using gp150^+^ and gp150^−^ versions of MuHV-4 made fluorescent by an eGFP tag on the abundant envelope component gM [38] (Fig. 4). Gp150^−^gM-eGFP^+^ virions bound better than gp150^+^gM-eGFP^+^ to all cell types, but the difference was most marked for HS^−^ CHO cells, A20 B cells and RAW-264 monocytes. Thus gp150^+^ virion binding correlated with cellular HS display, and gp150^−^ virion binding was strong regardless. Both gp150^−^ and gp150^+^ virions bound well to NS0 cells. Comparing Fig. 2 with Fig. 4 shows that MuHV-4 infected RAW-264 and HS^−^ CHO cells better than NS0 despite binding better to NS0 cells, and that gp150^−^ virions infected A20 cells poorly despite binding relatively well. These data therefore supported the idea of a post-binding block to B cell infection.

That gp150^−^ virions bound better than gp150^+^ to HS^+^ CHO cells (Fig. 4) but did not infect them better (Fig. 2) suggested that when cell binding was very strong, down-stream events could also limit epithelial infection. However they limited A20 cell infection even when HS expression (Fig. 3) and virion binding (Fig. 4) were weak. By comparison, HS^−^ CHO cells and RAW-264 cells showed a good correlation between better gp150^−^ virion binding and better infection. Therefore a post-binding restriction of infection was possible for any cell, but was much more severe for B cells.

### HS up-regulation confirms that B cell infection is blocked post-binding

**Figure 5:**
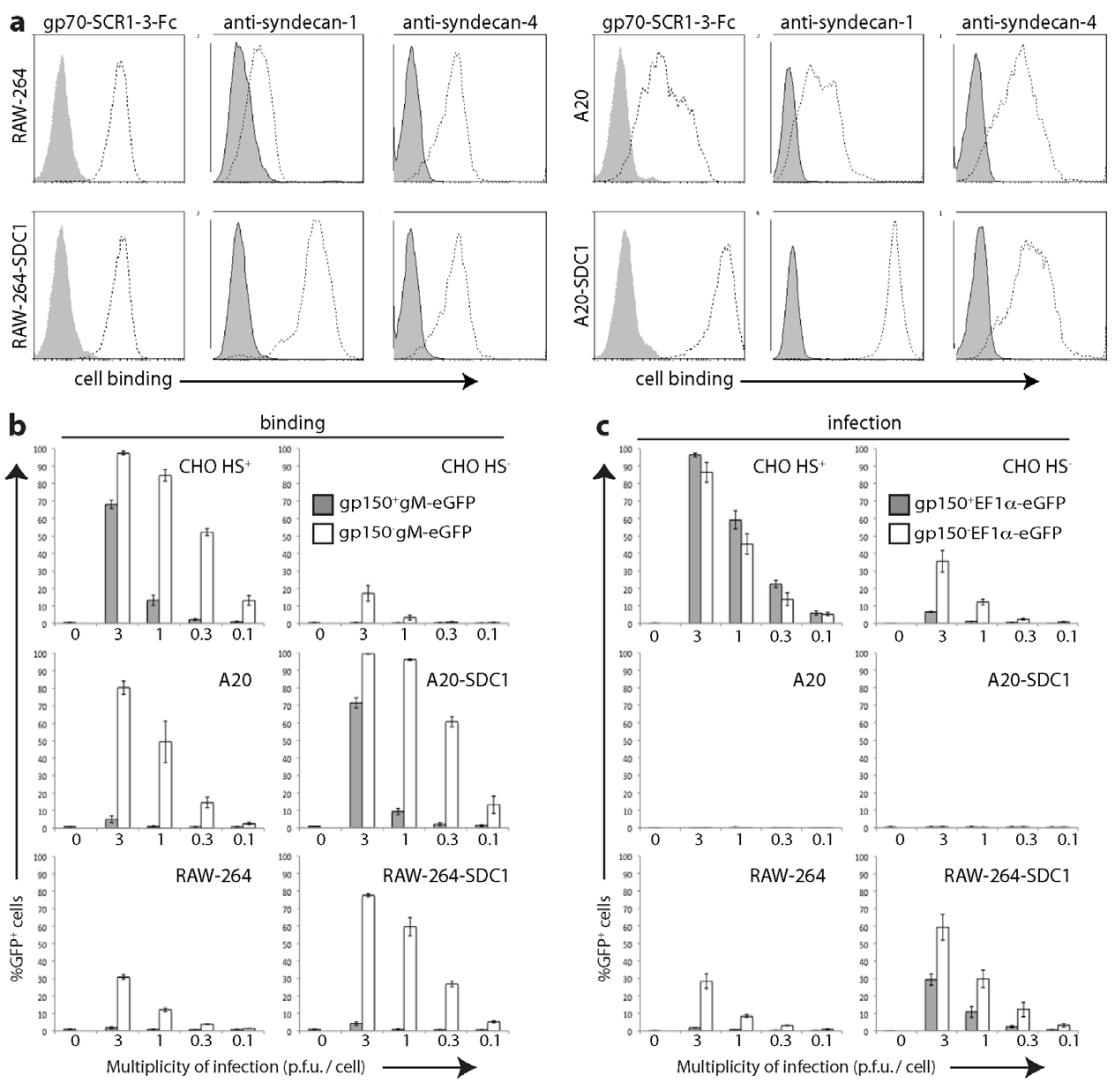
Effect of syndecan-1 up-regulation on cell binding and infection. **a.** RAW-264 monocytes and A20 B cells were transduced or not with an uncleavable form of the HS carrier syndecan-1 (SDC-1). Flow cytometry confirmed syndecan-1 up-regulation; syndecan-4 staining is shown for comparison. HS expression was assayed by staining with the gp70 HS binding domains linked to IgG-Fc (gp70-SCR1-3-Fc). Open histograms show specific staining; closed histograms show staining by secondary antibody alone. Equivalent data were obtained in a repeat experiment. **b.** Cells were exposed to gp150^+^ and gp150**^−^** versions of MuHV-4 with eGFP linked to the cytoplasmic tail of gM (gM-eGFP) (4 h, 37°C). After washing ×3 in PBS, 20,000 cells were assayed for viral eGFP uptake by flow cytometry. Bars show mean ± SEM of 3 replicate experiments. 0 = no virus. SDC-1 expression significantly increased gp150^+^ virion binding to A20 cells and gp150^−^ virion binding to both A20 and RAW-264 cells (p<0.01 by Student's t test for at least 2 virus dilutions). **c.** Cells were exposed to wild-type (gp150^+^) or gp150**^−^** versions of EF1α-eGFP MuHV-4 (18 h, 37°C). 20,000 cells were then assayed for viral eGFP expression by flow cytometry. Bars show mean ± SEM of 2 replicate experiments. 0 = no virus. SDC-1 expression significantly increased RAW-264 cell infection by gp150^+^ and gp150^−^ viruses (p<0.01 by Student's t test for at least 2 virus dilutions) but not A20 cell infection.

We tested further the relationship between cellular HS expression and MuHV-4 infection by expressing in RAW-264 and A20 cells an uncleavable form of the HS carrier syndecan-1 (SDC-1) (Fig. 5). This increased gp150^−^ as well as gp150^+^ virion binding, presumably because gp70 and gH/gL attach virions better than does just the HS-independent binding regulated by gp150. Gp70-SCR1-3-Fc bound only marginally better to RAW-264-SDC-1 cells than to RAW-264 (Fig. 5a), but virion binding (Fig. 5b) and infection (Fig. 5c) both increased. By contrast, both gp70-SCR1-3-Fc (Fig. 5a) and virions (Fig. 5b) bound substantially better to A20-SDC-1 cells than to A20, but infection remained negligible (Fig. 5c). Therefore again there was evidence of a post-binding block to B cell infection.

### Co-culture with infected fibroblasts improves myeloid infection but not B cell infection

**Figure 6:**
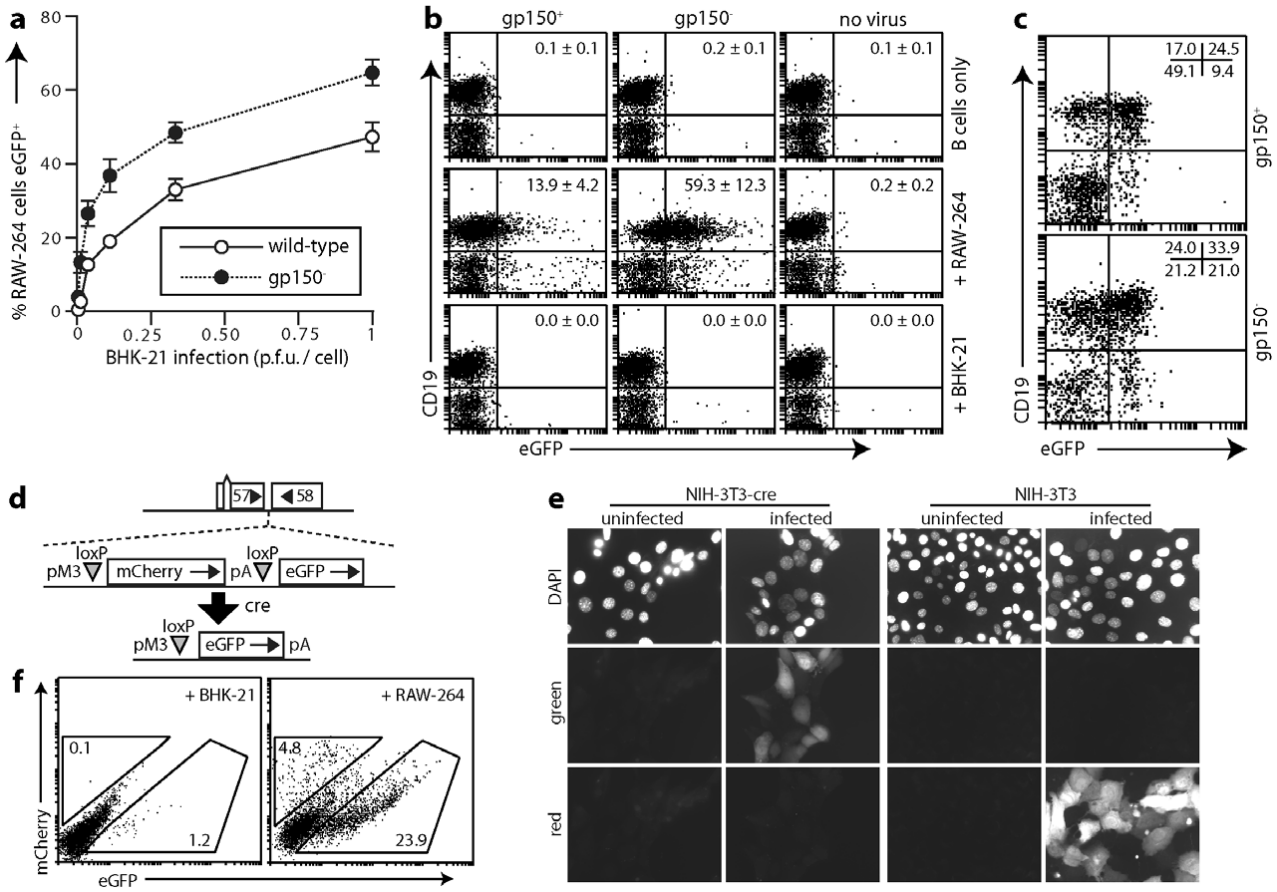
Infection by co-culture. **a.** BHK-21 cells were infected with gp150^+^ or gp150^−^ EF1α-eGFP MuHV-4 at different multiplicities (2 h, 37°C), then washed ×3 in PBS and cultured overnight with an equal number of RAW-264 cells. 18 h later RAW-264 cells (CD11b^+^) eGFP expression was assayed by flow cytometry. Each point shows the mean ± SD of 3 replicate cultures. Equivalent data were obtained in 3 experiments. **b.** Gp150^+^ or gp150^−^ EF1α-eGFP MuHV-4 (10^6^ p.f.u.) was added to 3×10^5^ RAW-264 cells or BHK-21 cells. 18 h later 10^6^ spleen cells were added to these cultures. As a control, a further 10^6^ spleen B cells were exposed directly to 10^6^ p.f.u. of virus. 24 h later eGFP expression in B cells (CD19^+^) was assayed by flow cytometry. The numbers show mean ± SD %eGFP^+^ of total CD19^+^ cells in 5 experiments. All BHK-21 cells and most RAW-264 cells were excluded from the flow cytometric analysis by FSC/SSC gating. The CD19^−^eGFP^+^ cells in RAW-264 cell co-cultures correspond to residual infected RAW-264 cells. The splenocytes included very few myeloid cells (<1% CD11b^hi^ or CD11c^+^). **c.** Gp150^+^ or gp150^−^ EF1α-eGFP MuHV-4 (10^6^ p.f.u.) was added to 10^6^ peritoneal macrophages. 3 days later, to allow for the limited lytic infection of these cells, we added 10^6^ spleen cells. 18 h later eGFP expression in B cells (CD19^+^) was assayed by flow cytometry. The numbers show the % cells in each quadrant. All CD19^−^eGFP^+^ cells were CD11b^hi^ macrophages. Again control BHK-21 cell co-cultures gave <0.5% eGFP^+^ B cells. Equivalent data were obtained in 3 experiments. **d.** A MuHV-4 recombinant (MHV-RG) was generated in which a viral M3 promoter (pM3) drives mCherry expression from the ORF57/ORF58 intergenic site. LoxP recombination by cre excises mCherry plus its polyadenylation signal to release eGFP expression from the same promoter. **e.** Cre^+^ or control cre^−^ NIH-3T3 cells were infected or not with MHV-RG (0.5 p.f.u./cell) and 18 h later imaged for red (mCherry) and green (eGFP) fluorescence. Nuclei were counterstained with DAPI. **f.** BHK-21 cells or RAW-264 cells were infected with MHV-RG (1 p.f.u./cell, 18 h) then co-cultured with cre^+^ NS0 cells. After 3 days the NS0 cells (MHC class II^hi^) were analysed for green and red fluorescence by flow cytometry. The percentage of total cells in each gate is indicated. Equivalent data were obtained in a repeat experiment.

Probably most *in vivo* herpesvirus spread occurs through cell/cell contacts rather than cell-free virion release [39]. Consistent with this idea, MuHV-4 lacking gp150 accordingly spreads normally *in vivo* despite poor virion release [27], whereas MuHV-4 lacking gp48, which is impaired in cell/cell spread, is attenuated [40]. Thus as B cells were infected down-stream of host entry (Fig. 1), we reasoned that their infection might involve cell/cell contact. However A20 B cells co-cultured overnight 1∶1 with infected BHK-21 cells (1 p.f.u./cell gp150^+^EF1α-eGFP^+^ or gp150^−^EF1α-eGFP^+^ virus, 24 h) remained <1% eGFP^+^ (0.10±0.02% eGFP^+^ for gp150^+^ and 0.31±0.04% eGFP^+^ for gp150^−^) (mean ± SD of triplicate cultures). By contrast RAW-264 monocytes co-cultured with infected BHK-21 cells became 40–60% eGFP^+^ (Fig. 6a). RAW-264 cells were infected approximately 30-fold better by cell-free gp150^−^ virions than by gp150^+^ (Fig. 2a); co-cultures showed only a 3-fold difference, presumably because cell/cell contact made virion binding less HS-dependent. Therefore direct contact with infected fibroblasts allowed efficient myeloid infection. The contrasting failure to improve B cell infection was consistent with this having an additional, post-binding block that required a different solution.

### B cell infection via myeloid cells

That MuHV-4 targets CD11c^+^ cells in lymph nodes [18] suggested that myeloid infection might make MuHV-4 B cell-tropic. We therefore next co-cultured A20 B cells 1∶1 with MuHV-4-exposed RAW-264 monocytes (3 p.f.u./cell, 18 h). In contrast to the negligible effect of co-culture with infected BHK-21 cells, this led to 6.15±1.28% of A20 cells becoming eGFP^+^ for gp150^+^ MuHV-4 and 12.79±2.23% becoming eGFP^+^ for gp150^−^ (mean ± SD of triplicate cultures). Co-culture with RAW-264 cells therefore promoted A20 B cell infection. Similar results were obtained with splenic B cells (Fig. 6b). We could also infect splenic B cells by co-culture with MuHV-4-infected peritoneal macrophages (Fig. 6c).

As a further measure of infection we used a MuHV-4 derivative (MuHV-RG) in which cre recombinase switches reporter gene expression: MuHV-RG is mCherry^+^ until it infects cre^+^ cells, when it becomes eGFP^+^ (Fig. 6d, 6e). Reporter gene expression is from a lytic cycle promoter, so as NS0 B cells support viral lytic gene expression [13] we co-cultured cre^+^ NS0 cells with MuHV-RG-exposed cre^−^ BHK-21 cells or cre^−^ RAW-264 cells. Co-culture with RAW-264 cells gave more eGFP^+^ NS0 cells than did co-culture with BHK-21 cells (Fig. 6f). Therefore myeloid infection promoted B cell infection.

### Myeloid-derived virions show enhanced B cell penetration

**Figure 7:**
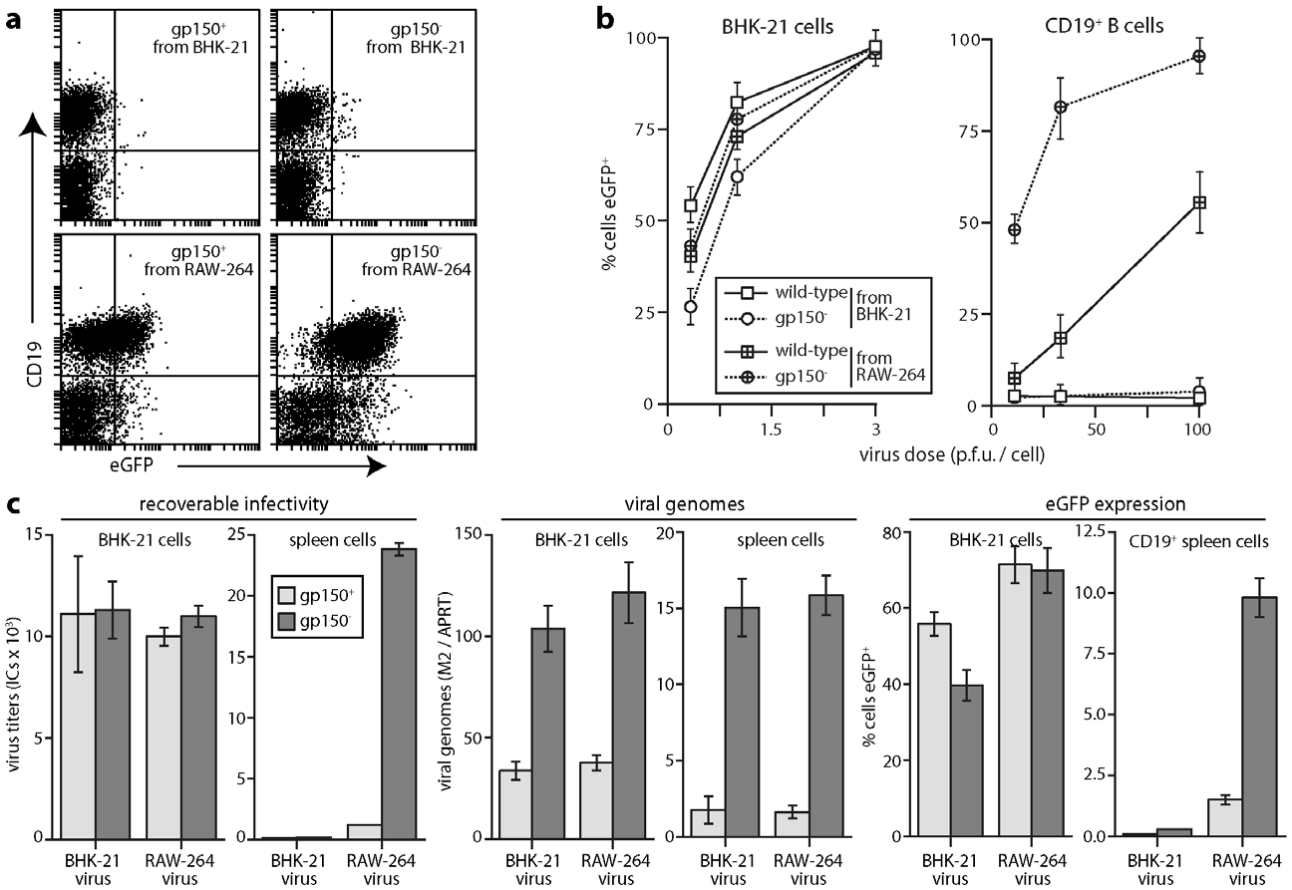
B cell infection by virions derived from RAW-264 cells. **a.** Gp150^+^ and gp150^−^ EF1α-eGFP virions were grown in BHK-21 fibroblasts or RAW-264 monocytes, then added (3 p.f.u./cell, 18 h) to spleen cells. An example flow cytometric plot of eGFP expression in CD19^+^ B cells is shown. Data from replicate experiments are summarized in **b**. **b.** Gp150^+^ or gp150^−^ EF1α-eGFP virions, propagated in BHK-21 or RAW-264 cells were added to BHK-21 cells or spleen cells at different multiplicities. 18 h later cells were scored as eGFP^+^ or eGFP^−^ by flow cytometry. Each point shows mean ± SD of 5 experiments. **c.** Gp150^+^ or gp150^−^ EF1α-eGFP virions from BHK-21 cells or RAW-264 cells were added to BHK-21 cells (0.5 p.f.u./cell) or to spleen cells (3 p.f.u./cell). After 4 h at 37°C, residual input virus was inactivated by acid wash. The cells were then split for analysis of recoverable infectivity by infectious centre assay, virus binding by Q-PCR of viral (M2) versus cellular (APRT) genome load and after further culture (16 h, 37°C) for flow cytometric assay of eGFP expression. Each bar shows mean ± SEM of triplicate cultures. Equivalent data were obtained in a repeat experiment.

Co-culture infections are complicated and so difficult to dissect further. We therefore tested next whether cell-free virions derived from myeloid cells could also infect B cells (Fig. 7). Gp150^+^ virions from RAW-264 cells gave greater eGFP expression in splenic B cells than did those from BHK-21 cells; RAW-264-derived gp150^−^ virions worked substantially better (Fig. 7a, 7b), indicating that without cell/cell contact gp150 significantly inhibited B cell binding. Viral DNA quantitation by Q-PCR (Fig. 7c) showed more gp150^−^ than gp150^+^ binding to B cells, with little difference between RAW-264 cell-derived and BHK-21 cell-derived virions. Infectious centre assays and eGFP expression by contrast showed considerably more infection by RAW-264 cell-derived virions. Therefore virion passage through myeloid cells improved B cell penetration rather than B cell binding.

### Antigenic changes in myeloid cell-derived virions

**Figure 8:**
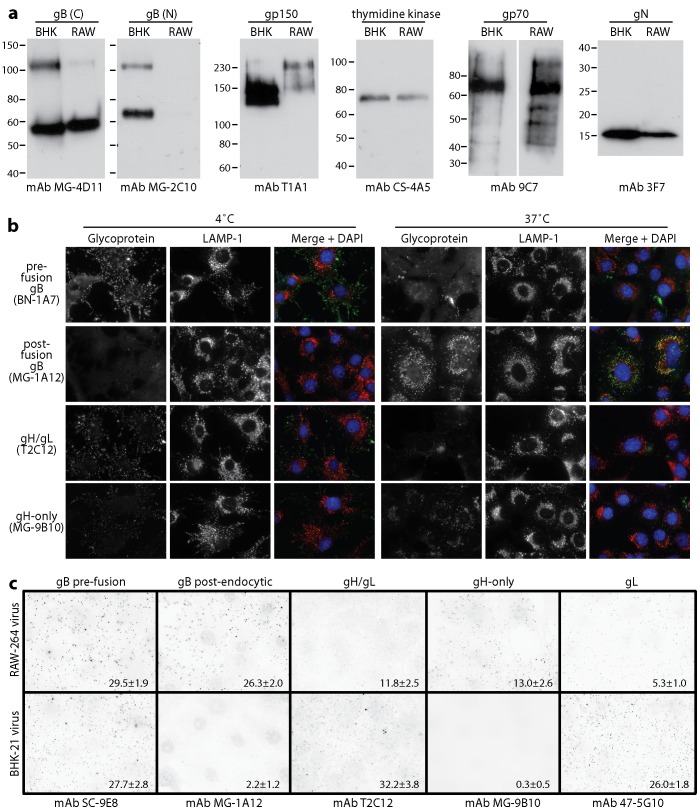
Glycoprotein analysis of MuHV-4 propagated in RAW-264 cells. **a.** EF1α-eGFP virions propagated in BHK-21 (BHK) or RAW-264 (RAW) cells were assayed for virion components by immunoblotting. gB (C) and gB (N) are the C-terminal and N-terminal halves of gB after its cleavage by furin. The upper band corresponds to uncleaved gB. **b.** Wild-type MuHV-4 virions propagated in BHK-21 cells were bound to NMuMG cell monolayers (3 h, 4°C, 3 p.f.u./cell), then washed ×2 in PBS and either fixed immediately, or incubated further (2 h, 37°C) before fixation to allow virion endocytosis. The cells were then analysed by immunofluorescence for virion antigen display. **c.** EF1α-eGFP virions propagated in BHK-21 or RAW-264 cells were bound to NMuMG cell monolayers (3 h, 4°C, 25 eGFP units/cell - equivalent to 3 p.f.u./cell), then washed ×2 in PBS, fixed and analysed by immunofluorescence for virion antigen display. Each dot corresponds to a positive virion. The numbers show mean ± SD positive virions per cell for 25 cells per sample. Comparison by Student's t test showed that significantly more RAW-264-derived virions than BHK-21 cell-derived virions were recognized by mAbs MG-1A12 and MG-9B10, and significantly fewer by mAbs T2C12 and 47-5G10 (p<0.01). Equivalent data were obtained in 2 further experiments.

Immunoblotting showed differences in gB and gp150 between BHK-21 cell-derived and RAW-264 cell-derived virions (Fig. 8a). However these seemed unlikely to account for their different tropisms: gB N-terminus recognition by mAb MG-2C10, which depends on cell type-specific O-glycosylation, was reduced for RAW-264 cell-derived virions but deleting the gB N-terminus has no obvious effect on B cell colonization [41]; gp150 migration and recognition, which also vary with O-glycosylation [42], were different between BHK-21 and RAW-264 cell-derived virions but gp150 disruption enhanced B cell binding by both without being sufficient for infection (Fig. 7).

An important feature of virions not revealed by immunoblotting is that gB and gH - which drive herpesvirus membrane fusion - change in antigenicity during cell entry [43], [44]. The gH of extracellular virions is bound to gL and so recognized by gH/gL-specific mAbs; following endocytosis gH/gL epitopes are lost and gH-only epitopes retained; upon fusion, which occurs in late endosomes, gH-only epitopes are also lost. Similarly, the gB of extracellular virions is recognized by mAb BN-1A7 but not mAb MG-1A12; following endocytosis gB gains MG-1A12 recognition; then upon fusion it loses BN-1A7 recognition [45]. Thus virions progress from BN-1A7^+^MG-1A12^−^gH/gL^+^gH-only^lo^ (extracellular) to BN-1A7^+^MG-1A12^+^gH/gL^−^gH-only^hi^ (post-endocytic, but still pre-fusion) to BN-1A7^−^MG-1A12^+^gH/gL^−^gH-only^−^ (post-fusion). This is illustrated in Fig. 8b: BHK-21 cell-derived virions bound to NMuMG cells at 4°C were not recognized by mAb MG-1A12 (post-endocytic gB) and were poorly recognized by mAb MG-9B10 (gH-only); after endocytosis, virions started to lose recognition by T2C12 and BN-1A7, strongly gained recognition by MG-1A12, and showed some increase in recognition by MG-9B10. The residual BN-1A7, T2C12 and MG-9B10 staining after 2 h at 37°C was outside LAMP-1^+^ late endosomes, that is on virions that had not yet reached their site of fusion.

RAW-264 cell-derived virions by contrast constitutively showed the post-endocytic forms of gB and gH (Fig. 8c). The mAb used here to detect pre-fusion gB - SC-9E8 - behaves the same as mAb BN-1A7 [45], and staining for gL followed the same pattern as gH/gL. Thus myeloid-derived virions appeared to penetrate B cells better because they were already primed for membrane fusion.

### *In vivo* evidence for virus passage through myeloid cells

**Figure 9:**
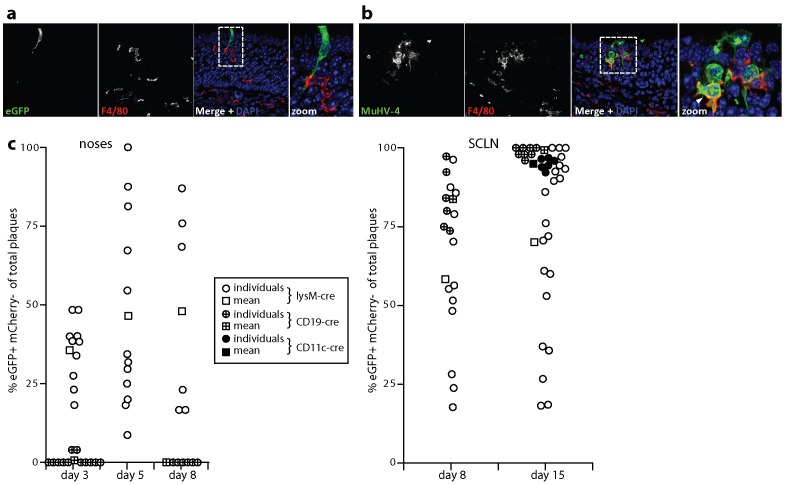
Tracking myeloid infection *in vivo*. **a.** C57BL/6 mice were infected i.n. with EF1α-eGFP^+^ MuHV-4 (3×10^4^ p.f.u. in 5 µl) and 1 day later analysed by immunofluorescent staining of the neuroepithelium for viral eGFP (green) and for macrophages with mAb F4/80 (red). The image shows a single infected epithelial cell - that is before substantial virus spread - contacting a macrophage. **b.** Mice were infected as in **a**, then analysed 1 day later for viral lytic antigen expression with an immune rabbit serum (green) and for macrophage distribution with mAb F4/80 (red). The arrowhead in the zoomed merge shows lytic antigens in an F4/80^+^ cell (yellow). **c.** Cre transgenic mice were infected i.n. with MHV-RG (3×10^4^ p.f.u. in 5 µl), and the recovered virus analysed for cre-mediated switching from red (eGFP^−^mCherry^+^) to green fluorescence (eGFP^+^mCherry^−^). Each point shows the % eGFP^+^mCherry^−^ of total plaques. Round points show individual mice, square points show means. Virus was recovered from noses by plaque assay at days 3, 5 and 8 post-infection for lysM-cre mice and at days 3 and 8 for CD19-cre mice. CD11c-cre mice were not analysed. Virus was recovered from the draining superficial cervical lymph nodes (SCLN), by infectious center assay at days 8 and 15 post-infection for lysM-cre and CD19-cre mice, and at day 15 for CD11c-cre mice. Plaques and infectious centers from non-transgenic C57BL/6 mice were 100% eGFP^−^mCherry^+^.

An important *in vivo* role for myeloid infection in B cell colonization would predict that it occurs early after host entry. We tested this by immunostaining acutely infected noses. At 1 day post-infection F4/80^+^ macrophages contacted viral eGFP^+^ epithelial cells (Fig. 9a), and infected macrophages occupied areas of viral lytic gene expression (Fig. 9b). Therefore myeloid infection immediately followed epithelial infection and preceded B cell infection (Fig. 1).

To identify whether the virus reaching B cells had previously replicated in a myeloid cell we infected with the MHV-RG floxed reporter virus lysM-cre mice, which express cre recombinase in macrophages, granulocytes, and some dendritic cells [46] (Fig. 9c). Virus recombination rates varied between mice, but at 3 days post-inoculation approximately 1/3 of the MHV-RG recovered from lysM-cre mouse noses was eGFP^+^mCherry^−^, indicating prior replication in a myeloid cell. At days 5 and 8 the mean recombination rate was 1/2. By contrast almost all the virus recovered from noses of CD19-cre mice, whch express cre recombinase in B cells [47], remained eGFP^−^mCherry^+^, consistent with B cells not being a primary infection target in the upper respiratory tract, and with myeloid infection preceding that of B cells.

B cells are the major site of MuHV-4 lymphoid infection [29], and virus recovered from the draining lymph nodes (SCLN) of CD19-cre mice was 80% eGFP^+^mCherry^−^ at day 8 and almost 100% eGFP^+^mCherry^−^ at day 15. LysM-cre mice again showed individual variation, but on average more than half the SCLN virus was eGFP^+^mCherry^−^. At day 15 post-infection we also assayed CD11c-cre mice, which express cre recombinase predominantly in dendritic cells [48]. Virus recovered from their SCLN was >90% eGFP^+^mCherry^−^. LysM and CD11c expression show only modest overlap [46]. Thus most if not all the virus reaching lymph nodes and infecting B cells appeared to have passed through at least one myeloid cell.

## Discussion

An enduring puzzle with MuHV-4 and KSHV has been that they persist in B cells *in vivo* yet infect them poorly *in vitro*. For MuHV-4 this is despite virions growing to high titers and efficiently infecting mice. We identified a binding block to myeloid infection that was overcome by co-culture, and a post-binding block to B cell infection that was overcome by virus propagation in myeloid cells. Better B cell penetration was associated with virions displaying a post-endocytic form of the gB/gH fusion complex. Consistent with these tropisms, host entry was an epithelial infection; myeloid infection followed; lymphoid infection occurred only later; and most if not all of the virus recovered from lymph nodes showed previous myeloid infection. Virion tropism therefore matched host antigen transport in setting an epithelial to myeloid to lymphoid infection cycle.

Virus binding also presented a hurdle to B cell infection. This increased when B cell HS expression increased. However while plasma cell differentiation upregulates B cell HS [49], MuHV-4 persists in memory rather than plasma B cells; and while interferon upregulates B cell HS [50] it also inhibits MuHV-4 infection. Therefore *in vivo* B cell binding seems unlikely to depend on HS up-regulation. Binding also increased when virions lacked gp150, but whether such virions are naturally produced is unclear: in contrast to the Bovine Herpesvirus-4 gp180 [51], RT-PCR has not demonstrated gp150 truncation by splicing (unpublished data); nor were myeloid cell-derived virions functionally gp150-deficient. The relative gp150-independence of B cell infection by co-culture suggested that binding occurs instead through myeloid/B cell contact [52]. Myeloid infection in turn probably involves contact with infected epithelial cells. Thus a need for HS binding by cell-free virions applies mainly to host entry [53], where the target was epithelial.

Penetration presented a more severe block to B cell infection. B cell penetration by EBV requires its gp42 [54]. MuHV-4 and KSHV lack obvious gp42 homologs and no MuHV-4 glycoprotein knockout has specifically failed to infect B cells [24], [40], [53], [55]–[57]. Therefore a B cell-specific component to the MuHV-4 fusion complex, while difficult to exclude, seems unlikely. Instead B cell-tropic virions showed antigenic changes in gB and gH. These corresponded to post-endocytic conformation changes that normally precede epithelial membrane fusion [45]. If these conformation changes are a prerequisite for fusion and are not triggered by B cells, it may be crucial that they are triggered by virion exit through an endocytic/exocytic compartment of myeloid cells. Simple co-culture with macrophages increased B cell infection at least 100-fold; specific lymphoid architecture and myeloid/B cell communication pathways doubtless make the process more efficient still *in vivo*.

Most *in vivo* myeloid populations are heterogeneous, but classically dendritic cells move from peripheral sites to lymph nodes, whereas macrophages are sessile. Thus dendritic cells could play an important role in virus transport. Alternatively, lymphatic virion transport could connect roles for macrophages in the periphery, where F4/80^+^ cells were closely associated with epithelial infection, and in lymph nodes, where viral DNA is found in CD11b^+^CD11c^−^ cells [18]. CD11c^+^ cells are mainly dendritic and lysM^+^ cells mainly macrophages. Some macrophages express CD11c [58], [59] and some CD11c^+^ cells express lysM, but the overlap is not extensive and not even all macrophages express lysM [46]. Thus the high percentages of MHV-RG recombination in both CD11c-cre and lysM-cre mice suggested that MuHV-4 might infect both macrophages and dendritic cells before reaching B cells.

The epithelial/myeloid/lymphoid MuHV-4 infection pathway is quite different to the epithelial cell/B cell exchange proposed for EBV [60]. Thus despite rhadinoviruses and lymphocryptoviruses colonizing similar cell populations, they may do so in different ways. Epithelial and fibroblast-derived MuHV-4 are strongly epithelial-tropic - we find little difference between epithelial and fibroblast infections [55] - as are NS0 cell-derived virions (data not shown); and even RAW-264 cell-derived MuHV-4 infected epithelial cells better than B cells. This consistent epithelial tropism perhaps reflects a predominant need for epithelial entry in the viral lifecycle: new infected B cells can come from lymphoproliferation, but each new epithelial infection likely requires new cell binding and penetration. EBV may have evolved ways to minimise its need for new epithelial infections. However it does not present clinically until infection is well established, so events equivalent in timing to the epithelial and myeloid infections of MuHV-4 are rarely studied. The striking parallels between MuHV-4 host colonization and normal antigen transport would suggest that other lymphotropic viruses follow similar routes.

## Materials and Methods

### Ethics statement

All animal experiments were approved by the University of Cambridge ethical review board and by the UK Home Office under the 1986 Animal (Scientific Procedures) Act as Project Licence 80/2538.

### Mice

C57BL/6 (Harlan UK), LysM-cre [46], CD19-cre [47] and CD11c-cre mice [48] were housed in the Cambridge University Department of Pathology animal unit. LysM-cre mice express cre recombinase in monocyte/macrophages and neutrophils in place of myeloid-specific lysozyme, CD19-cre mice express cre in B cells, and CD11c-cre mice express cre predominantly in dendritic cells. Mice were given MuHV-4 intranasally (i.n.) either in 30 µl under general anesthesia to infect both the upper and lower respiratory tract, or in 5 µl without anesthesia to infect just the upper respiratory tract.

### Cells

BHK-21 fibroblasts (American type culture collection CCL-10), CHO-K1 epithelial cells (CCL-61), the glycosaminoglycan-deficient CHO-745 mutant (CRL-2242), NMuMG epithelial cells (CRL-1636), NS0 myeloma cells, A20 B cells (TIB-208), NIH-3T3 cells (CRL-1658), NIH-3T3-cre cells [61] 293T cells (CRL-11268), and RAW-264 monocytes (TIB-71) were cultured in Dulbecco's Modified Eagle's Medium with 2 mM glutamine, 100 U/ml penicillin, 100 mg/ml streptomycin and 10% fetal calf serum. Primary cells were cultured in the same medium supplemented with 50 mM 2-mercaptoethanol. Peritoneal macrophages were harvested 5 days after intraperitoneal injection of Brewer's thioglycollate medium (Sigma Chemical Co.), by injecting and aspirating post-mortem 10 ml of Dulbecco's Modified Eagle's Medium. Cells non-adherent to tissue culture plates (Nunc) after 2 h at 37°C were discarded. The remaining cells were routinely >90% CD11b^hi^CD19^−^ by flow cytometry. Spleens were disrupted into single cell suspensions by homogenization in a Griffiths tube. Debris was removed by filtration (200 µm), and erythrocytes and dead cells were removed by centrifugation on Ficoll. The cells recovered were typically 60% B cells CD19^+^, 25% CD4^+^ T cells and 15% CD8^+^ T cells. <1% of the recovered cells were macrophages (CD11b^hi^) or dendritic cells (CD11c^+^). Retroviral transduction of A20 cells with an uncleavable form of the syndecan-1 extracellular domain has been described [30]. We used the same approach to over-express syndecan-1 in RAW-264 cells, selecting transduced cells with Zeocin (Invitrogen). As with NIH-3T3-cre cells, we generated NS0-cre cells by transduction with a cre expressing retrovirus and selected transduced cells with G418.

### Viruses

All viruses were generated from a BAC-cloned MuHV-4 genome [62]. Gp150^+^ and gp150^−^ versions of MuHV-4 with a C-terminal eGFP tag on the abundant virion envelope component gM have been described [38], as has the generation of MuHV-4 with an intergenic EF1α-eGFP expression cassette [63]. We made a gp150^−^ version of the EF1α-eGFP BAC by RecA-mediated recombination of a genomic clone containing multiple stop codons at genomic co-ordinate 69743 [24]. This terminated the 483 amino acid gp150 coding sequence after 93 amino acids.

To make MuHV-4-RG, in which cre recombinase switched reporter gene expression from mCherry to eGFP, we started with a derivative of pEGFP-C2 (Clontech) in which the eGFP coding sequence had been replaced by that of mCherry [64]. We excised most of the polylinker by digesting with *Bam*HI + *Bgl*II, gel purifying, and religating, then PCR-amplified the mCherry coding sequence plus the downstream SV40 polyadenylation site with primers adding an outer *Eco*RI restriction site and an altered loxP site (loxP*) to each flank. The loxP* spacer region was GGATACTT rather than GCATACAT to make it incompatible with the loxP sites flanking the MuHV-4 BAC cassette. EGFP expression from an intergenic MuHV-4 M3 promoter (pM3-eGFP-pA) has been described [65]. We cloned the *Eco*RI-restricted loxP*-mCherry-pA-loxP* PCR product into the *Eco*RI site between the M3 promoter and the eGFP start codon of pM3-eGFP-pA in pSP73. Genomic flanks for recombination were then added by blunt end cloning pM3-loxP*-mCherry-pA-loxP*-eGFP-pA into the *Mfe*I site (genomic coordinate 77176) of a *Bgl*II genomic clone (75338–78717) in pSP73, then subcloning as a *Bgl*II fragment into the *Bam*HI site of the KanR^+^SacB^+^ori^ts^ shuttle vector pST76K-SR. pM3-loxP*-mCherry-pA-loxP*-eGFP-pA was then recombined into the MuHV-4 BAC by transient RecA expression, selection with kanamycin, and counter-selection with sucrose [62]. Recombinant clones were identified and checked for genomic integrity by restriction enzyme mapping. Infectious virus (mCherry^+^ from the reporter construct and eGFP^+^ from the BAC cassette) was recovered by transfecting BAC DNA into BHK-21 cells. This was then passed once through NIH-3T3-cre cells (infection at 2 p.f.u./cell). Viruses excising the BAC cassette but retaining the complete loxP*-flanked reporter cassette (eGFP^−^mCherry^+^) were selected from the mixed progeny by flow cytometric sorting of infected cells and cloning on BHK-21 cells. Correct insertion of the expression cassette was confirmed by viral DNA sequencing.

Viruses were grown in BHK-21 cells by low multiplicity infection (0.01 p.f.u./cell) and culture until >50% of cells showed cytopathic effects - typically 3–5 days. Viruses were grown in RAW-264 cells, which support lytic propagation less well, by infection at 0.1 p.f.u./cell and culture for 2–3 weeks, collecting the medium every 3–4 days and sub-culturing the cells as required. By this time >50% of the cells showed cytopathic effects. Virions were harvested from infected cell supernatants by ultracentrifugation (35,000× *g*, 90 min). Cell debris was removed by low speed centrifugation (500× *g*, 10 min) and by filtration (0.45 µm).

### Plasmids

Expression constructs for the N-terminal 3 short consensus repeats of gp70 and the N-terminal 250 amino acids of gp150, each fused to human IgG-Fc, have been described [37]. These were transfected into 293T cells using Fugene-6 (Roche Diagnostic Ltd). Recombinant proteins were collected from cell supernatants. The amounts of each Fc fusion were quantitated by immunoblotting for IgG-Fc and normalized on this basis.

### Virus assays

Tissue blocks containing the whole upper respiratory tract epithelium [64] were homogenized in a pestle and mortar; lungs were homogenized mechanically (Omni International). Virus titers were then determined by plaque assay [24]. BHK-21 cell monolayers were incubated with virus dilutions (2 h, 37°C), overlaid with 0.3% carboxymethylcellulose and 4 days later fixed with 4% formaldehyde and stained with 0.1% toluidine blue for plaque counting. Virus titers in spleens and lymph nodes were determined by infectious centre assay of single cell suspensions [24]. Tissue samples from mice infected with MHV-RG were plated at limiting dilution and positive wells scored for red or green fluorescence under UV illumination.

Viral genome loads were measured by Q-PCR [64]. MuHV-4 genomic co-ordinates 4166–4252 (M2 gene) was amplified (Rotor Gene 3000, Corbett Research) from 50 ng DNA extracted from *ex vivo* organs (Promega Corporation). The PCR products were quantitated by hybridization with a Taqman probe (genomic coordinates 4218–4189) and converted to genome copies by comparison with a standard curve of cloned plasmid template, amplified in parallel. Cellular DNA was quantitated in the same reaction by amplifying part of the adenosine phosphoribosyl transferase (APRT) gene, again with Taqman probe hybridization and known template dilutions amplified in parallel for quantitation. Virus loads were then normalized by the cellular genome copy number of each sample.

### Flow cytometry

Green (eGFP) and red (mCherry) fluorescence were measured directly. For antibody staining, adherent cells were plated overnight onto Petri dishes, then detached without trypsinization. Non-adherent cells were used directly. B cells were identified by staining with a phycoerythrin-conjugated rat mAb to CD19 (BD Biosciences) or an Alexafluor633-conjugated pAb to mouse immunoglobulin (Invitrogen). Syndecan-1 and syndecan-4 were detected with phycoerythrin-conjugated mAbs (BD Biosciences); heparan sulfate was detected with mAbs F58-10E4 and NAH46 (Seikagaku Corporation) plus Alexafluor488-conjugated pAb to mouse immunoglobulin (Invitrogen). Analysis was performed on a FACS Calibur and sorting on a FACS Vantage (BD Biosciences).

### Protein analysis

Virions were denatured by heating in Laemmli's buffer (50°C, 10 min), then proteins resolved by SDS-PAGE and either stained with Coomassie Brilliant Blue followed by destaining in acetic acid/methanol, or transferred to PVDF membranes. The membranes were blocked in 10% non-fat milk then incubated with mAbs specific for the gB N-terminus (MG-2C10) [41], the C-terminal half of gB (MG-4D11) [43], gp70 (9C7) [33], gN (3F7) [66], gp150 (T1A1) [24] and thymidine kinase (CS-4A5) [67]. EGFP was detected with a rabbit pAb (Abcam). Antibody binding was detected with horseradish peroxidase-conjugated rabbit anti-mouse IgG pAb or donkey anti-rabbit IgG pAb (Dako Corporation). Development was with ECL reagents (APBiotech) and exposure to X-ray film.

### Immunofluorescence

To analyse virion antigenicity, NMuMG cells were adhered to glass coverslips. Virions were then added to the cells at 4°C to allow binding but not endocytosis. The cells were then fixed (2% formaldehyde, 30 min, 4°C) and blocked in PBS/5% fetal calf serum/0.1% Tween-20. Pre-endocytic gB was detected with mAbs SC-9E8 or BN-1A7 [45], post-endocytic gB with mAb MG-1A12 [43], gH/gL with mAb T2C12 [55], gL with mAb 47-5G10 [68] and gH-only with mAb MG-9B10 [55]. In some experiments LAMP-1 was detected with mAb 104B (BD Biosciences). After the primary antibody incubations (1 h, 23°C) the cells were washed ×3 in PBS/0.1% Tween-20, incubated with Alexafluor 568-coupled goat anti-mouse-IgG pAb or with Alexafluor 488-coupled goat anti-mouse-IgG pAb + Alexafluor 568-coupled goat anti-rat IgG pAb (1 h, 23°C) (Invitrogen), washed ×3 in PBS/0.1% Tween-20, mounted in Prolong Gold with DAPI (Invitrogen), and imaged with an Olympus microscope plus Hamamatsu digital camera or with a Leica SP2 confocal microscope.

### Immunohistochemistry

The anterior part of the skull containing the olfactory epithelium was removed post-mortem and fixed in 4% formaldehyde–PBS (4°C, 24 h). Samples were decalcified in 250 mM EDTA (two weeks, 23°C, changing the buffer every 2–3 days), then washed ×2 in PBS and paraffin-embedded. 7 µm sections were cut, de-paraffinised in xylene and rehydrated in ethanol/water. Antigen retrieval was performed by microwaving (850W, 5 min) in 10 mM NaCitrate pH 6/0.05% Tween-20. Endogenous peroxidase activity was quenched in PBS/3% H_2_O_2_ for 10 min. The sections were then blocked with 2% rabbit serum and viral antigens detected with a MuHV-4-immune rabbit serum (18 h, 23°C) [27], biotinylated goat anti-rabbit IgG pAb and Vectastain Elite ABC Peroxidase system with ImmPACT DAB substrate (Vector Laboratories), washing ×3 in PBS between each step. The sections were counterstained with Mayer's Hemalum (Merck) and mounted in DPX (BDH). Viral miRNA/tRNAs 1–4 were detected by *in situ* hybridisation [28]. After de-waxing in xylene and rehydration in ethanol/water, fixed sections were treated with proteinase K (100 µg/ml, 10 min, 37°C) and acetylated with 25% acetic anhydride in 0.1 M triethanolamine. They were then hybridized in 50% formamide/10 mM Tris pH 7.5 with a digoxigenin-labelled riboprobe, generated by T7 transcription of pEH1.4 (58°C, 18 h). Hybridized probe was detected with alkaline phosphatase-conjugated anti-digoxigenin Fab fragments (Boehringer Ingelheim) and BCIP/NBT substrate.

For fluorescence imaging, samples were fixed in 1% formaldehyde/10 mM sodium periodate/75 mM L-lysine (4°C, 24 h), equilibrated in 30% sucrose (4°C, 18 h), then frozen in OCT and sectioned (7 µm) on a cryostat. Sections were air dried (2 h, 23°C) and blocked with 2% serum/2% BSA/PBS (1 h, 23°C). We detected macrophages with mAb F4/80 (Serotec) plus Alexafluor568-conjugated goat anti-rat IgG pAb (Invitrogen). We detected viral eGFP expression with rabbit anti-eGFP pAb (Abcam) plus Alexafluor488-conjugated goat anti-rabbit IgG pAb (Invitrogen). Sections were washed ×3 in PBS after each antibody incubation (1 h, 23°C), then mounted in Prolong Gold + DAPI (Invitrogen), visualised using a Leica TCS SP2 confocal microscope, and analysed with ImageJ.

## Supporting Information

Figure S1 **Detection of B cell infection by viral eGFP expression.** **a.** An EF1α promoter was used to drive eGFP expression from the MuHV-4 ORF57/ORF58 intergenic site. **b.** C57BL/6 mice were infected intranasally (10^4^ p.f.u., 30 µl) with wild-type (WT) or EF1α-eGFP MuHV-4. Lungs and noses were titered by plaque assay after 5 days; superficial cervical lymph nodes (SCLN) and spleens were titered by infectious center (IC) assay after 14 days. Each point shows the titer of 1 mouse. Crosses show means. Student's t test showed no significant attenuation of EF1α-eGFP MuHV-4 in any site (p>0.2). **c.** Mice were infected as in **b** and 10 days later analysed by flow cytometry for eGFP expression in SCLN. Most eGFP^+^ cells were CD19^+^. The percentage of total CD19^+^ cells in the boxed region is indicated. Each plot shows the result for 1 mouse. Equivalent results were obtained in 2 further experiments. **d.** A20 cells over-expressing an uncleavable form of syndecan-1 were infected (5 p.f.u./cell) with MuHV-4 expressing eGFP from either an HCMV IE1 or EF1α promoter. 24 h later eGFP expression was analysed by flow cytometry. The percentage of total cells in the boxed region is indicated. Equivalent results were obtained in 5 experiments. **e.** The eGFP^+^ cells in **d** were enriched (to approximately 50%) by flow cytometric sorting and maintained for a further 10 days before re-analysing eGFP expression.
(TIF)

Figure S2 **Protein content per pf.u. of gp150^+^ and gp150^−^ viruses used in infectivity assays.** Aliquots of the gp150^+^ and gp150^−^ virus stocks used for infectivity assays were denatured by heating in Laemmli's buffer, then resolved by SDS-PAGE and stained with Coomassie Brilliant Blue. This established that equivalent numbers of p.f.u. contained equivalent amounts of viral protein, and so that one virus stock did not have an excess of non-infectious virions or contaminating cellular debris. Gp150 itself is difficult to identify by this means because it co-migrates with the abundant virion components ORF75c and ORF75b.
(TIF)

Figure S3 **Protein and eGFP content per pf.u. of gp150^+^ and gp150^−^ viruses used in binding assays.** Aliquots of the gp150^+^ and gp150^−^ virus stocks used for binding assays were denatured by heating in Laemmli's buffer, then resolved by SDS-PAGE and either stained with Coomassie Brilliant Blue (left panel) or immunoblotted for eGFP with a polyclonal rabbit serum (right panel). This established that equivalent numbers of p.f.u. contained equivalent amounts of viral protein and gM-eGFP, and so that one virus stock did not have an excess of non-infectious virions or contaminating cellular debris. The higher bands on the immunoblot are likely to be aggregated gM-eGFP, since gM tends to precipitate when excessively denatured.
(TIF)
