# Supplementary figures and images for: Myeloid Infection Links Epithelial and B Cell Tropisms of Murid Herpesvirus-4
Source: PLoS Pathog. 2012 Sep 20;8(9):e1002935. doi: 10.1371/journal.ppat.1002935 (PMC3447751; doi:10.1371/journal.ppat.1002935)

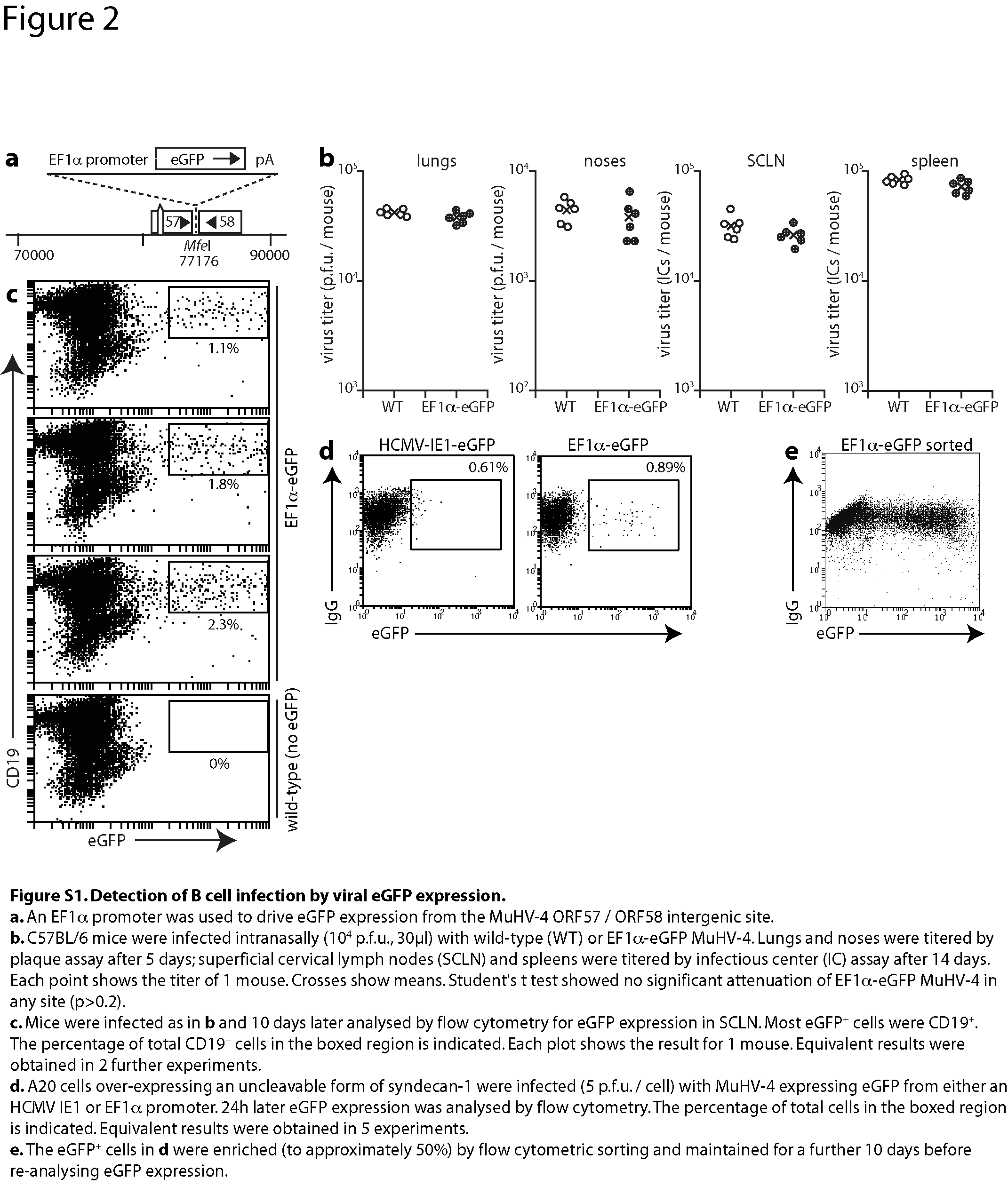

Supplement: Figure S1 — Detection of B cell infection by viral eGFP expression. a. An EF1α promoter was used to drive eGFP expression from the MuHV-4 ORF57/ORF58 intergenic site. b. C57BL/6 mice were infected intranasally (104 p.f.u., 30 µl) with wild-type (WT) or EF1α-eGFP MuHV-4. Lungs and noses were titered by plaque assay after 5 days; superficial cervical lymph nodes (SCLN) and spleens were titered by infectious center (IC) assay after 14 days. Each point shows the titer of 1 mouse. Crosses show means. Student's t test showed no significant attenuation of EF1α-eGFP MuHV-4 in any site (p>0.2). c. Mice were infected as in b and 10 days later analysed by flow cytometry for eGFP expression in SCLN. Most eGFP+ cells were CD19+. The percentage of total CD19+ cells in the boxed region is indicated. Each plot shows the result for 1 mouse. Equivalent results were obtained in 2 further experiments. d. A20 cells over-expressing an uncleavable form of syndecan-1 were infected (5 p.f.u./cell) with MuHV-4 expressing eGFP from either an HCMV IE1 or EF1α promoter. 24 h later eGFP expression was analysed by flow cytometry. The percentage of total cells in the boxed region is indicated. Equivalent results were obtained in 5 experiments. e. The eGFP+ cells in d were enriched (to approximately 50%) by flow cytometric sorting and maintained for a further 10 days before re-analysing eGFP expression. (TIF) [file ppat.1002935.s001.tif]

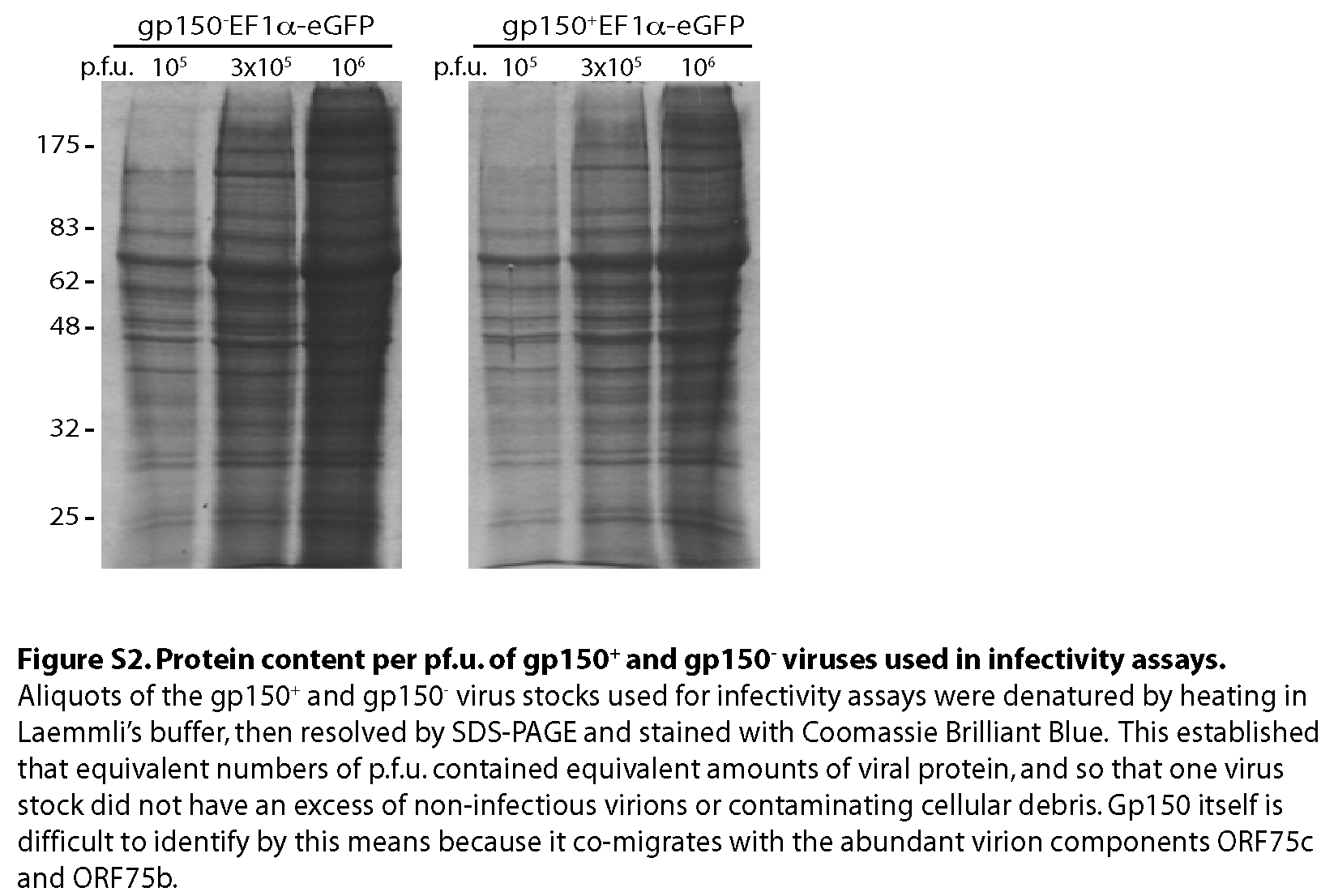

Supplement: Figure S2 — Protein content per pf.u. of gp150+ and gp150− viruses used in infectivity assays. Aliquots of the gp150+ and gp150− virus stocks used for infectivity assays were denatured by heating in Laemmli's buffer, then resolved by SDS-PAGE and stained with Coomassie Brilliant Blue. This established that equivalent numbers of p.f.u. contained equivalent amounts of viral protein, and so that one virus stock did not have an excess of non-infectious virions or contaminating cellular debris. Gp150 itself is difficult to identify by this means because it co-migrates with the abundant virion components ORF75c and ORF75b. (TIF) [file ppat.1002935.s002.tif]

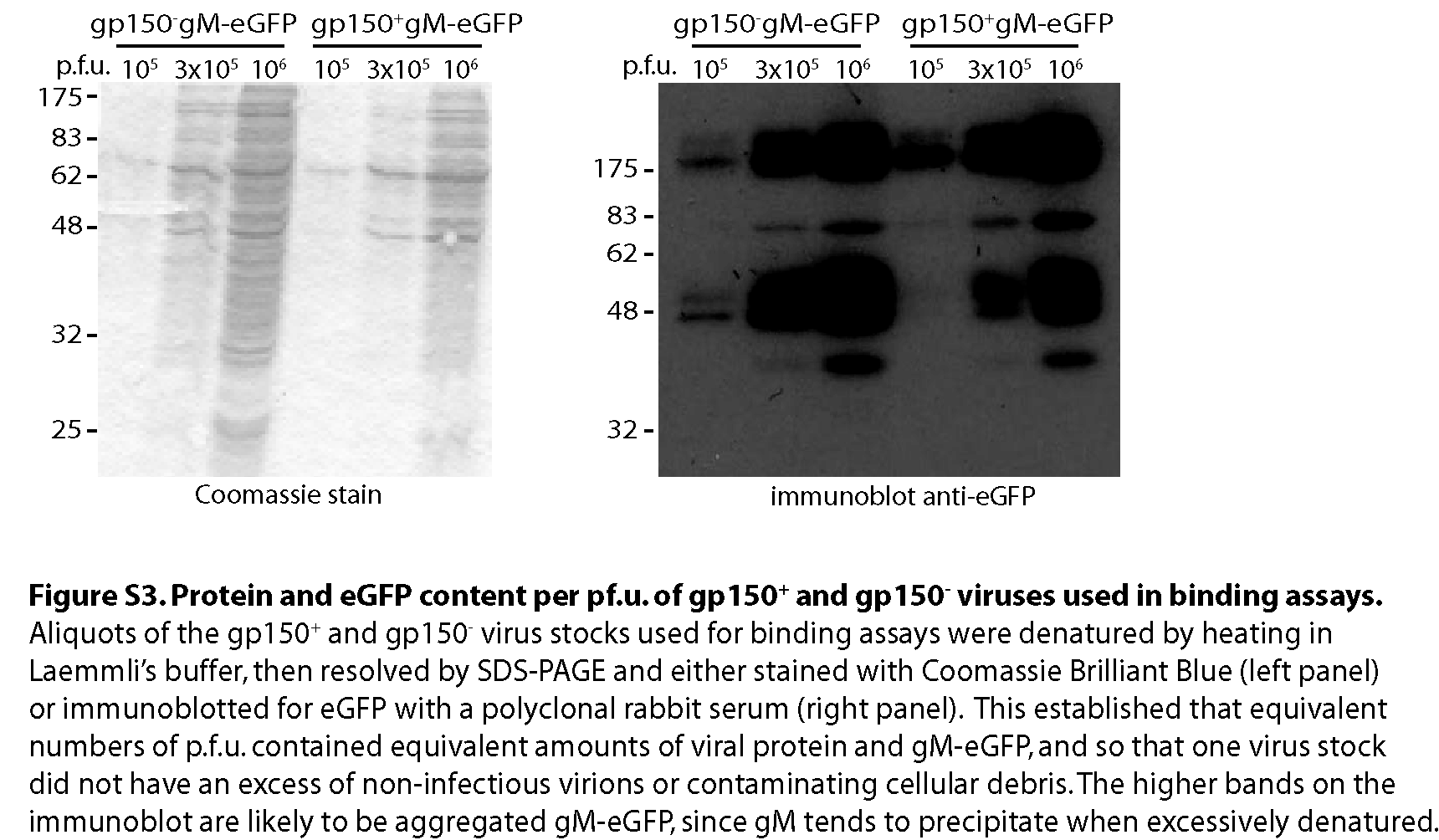

Supplement: Figure S3 — Protein and eGFP content per pf.u. of gp150+ and gp150− viruses used in binding assays. Aliquots of the gp150+ and gp150− virus stocks used for binding assays were denatured by heating in Laemmli's buffer, then resolved by SDS-PAGE and either stained with Coomassie Brilliant Blue (left panel) or immunoblotted for eGFP with a polyclonal rabbit serum (right panel). This established that equivalent numbers of p.f.u. contained equivalent amounts of viral protein and gM-eGFP, and so that one virus stock did not have an excess of non-infectious virions or contaminating cellular debris. The higher bands on the immunoblot are likely to be aggregated gM-eGFP, since gM tends to precipitate when excessively denatured. (TIF) [file ppat.1002935.s003.tif]
